# HOTAIR mediates cisplatin resistance in nasopharyngeal carcinoma by regulating miR-106a-5p/SOX4 axis

**DOI:** 10.1080/21655979.2022.2038429

**Published:** 2022-02-28

**Authors:** Wei Cao, Yi Sun, Long Liu, Junwei Yu, Jiabiao Ji, Yatang Wang, Jianming Yang

**Affiliations:** Department of Otorhinolaryngology, Head and Neck Surgery, The Second Hospital of Anhui Medical University, Hefei, China

**Keywords:** HOTAIR, miR-106a-5p/SOX4 axis, cisplatin resistance, nasopharyngeal carcinoma

## Abstract

This study explored the function and mechanisms of HOX transcript antisense RNA (HOTAIR) in the drug resistance of nasopharyngeal carcinoma (NPC). Quantitative PCR, Western blotting, MTT assay, flow cytometry, Transwell assay, and luciferase assay were performed. HOTAIR expression levels were upregulated in cisplatin (DDP)-resistant NPC tissues and cells. Knockdown of HOTAIR in DDP-resistant NPC cells increased cell sensitivity of DDP, as well as decreased cell viability, expression of chemoresistance-related proteins, migration and invasion, increased cell apoptosis. In addition, downregulation of microRNA 106a-5p (miR-106a-5p) expression and upregulation of SRY-box transcription factor 4 (SOX4) expression were observed in DDP-resistant NPC tissues and cells. MiR-106a-5p targets HOTAIR and SOX4; thus, silencing of HOTAIR significantly increased miR-106a-5p expression. The overexpression of miR-106a-5p significantly reversed the increase in SOX4 expression induced by HOTAIR lentivirus (Lv-HOTAIR). Knockdown of SOX4 reduced the drug resistance of DDP caused by the silencing of miR-106a-5p expression. In summary, HOTAIR enhanced DDP resistance in NPC cells by regulating the miR-106a-5p/SOX4 axis.

## Graphical abstract

**Figure:**
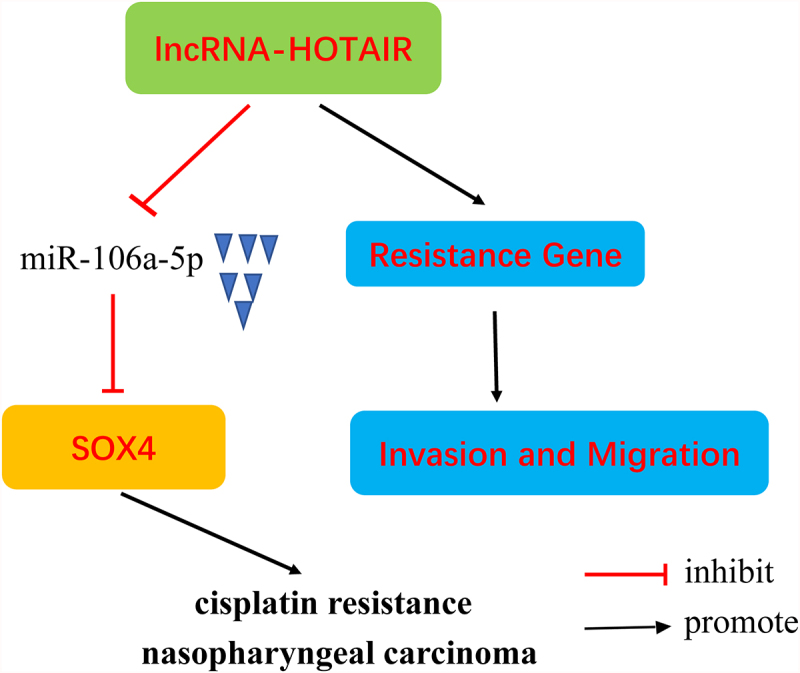

## 1. Introduction

Nasopharyngeal carcinoma (NPC) is a highly malignant neoplasm originating from nasopharyngeal mucosa [1]. The majority of patients diagnosed with NPC is between 40 and 60 years of age. The age-specific incidence of NPC in China is relatively high among the elderly population, and the age-specific incidence in males is significantly higher than in females [2]. At present, NPC is usually treated in various of ways, including radiotherapy, surgery, chemotherapy, targeted therapy, and immunotherapy [3]. However, chemotherapeutic drug resistance remains a big challenge [4]. Therefore, it is urgent to decipher and explore the underlying molecular mechanisms of drug resistance of NPC and to find novel therapeutic targets.

Long non-coding RNAs (lncRNAs) is a type of non-coding RNA molecule with a transcript length of more than 200 nucleotides, and together with short-chain non-coding RNA, it forms a non-coding RNA population [5,6]. The expression of lncRNAs is developmentally regulated, and shows tissue and cell specificity [7]. LncRNAs perform important biological functions. The lncRNA HOX transcript antisense RNA (HOTAIR) is abnormally expressed in a variety of cancers [8], such as breast cancer [9], lung cancer [10], and hepatocellular carcinoma (HCC) [11]. In addition, HOTAIR mediates cisplatin (DDP)-resistant non-small cell lung cancer by regulating the Wnt signaling pathway [12]; however, the role of HOTAIR in DDP resistance in NPC is not clearly understood.

MicroRNAs (miRNAs), approximately 22 nucleotides in length, could target the post-transcriptional level of the gene [13]. And miRNAs regulate many cellular pathways and functions including cell proliferation, differentiation, apoptosis, and ontogenesis [14,15]. Moreover, present studies have shown that miRNAs play key regulatory roles in drug resistance in many cancers [16]. MiR-106a-5p is abnormally expressed in many cancer types, including NPC [17], HCC [18], and colon cancer [19]. SRY-box transcription factor 4 (SOX4) has important biological functions including transcriptional regulation, intracellular material transport, and chromosome remodeling, and participates in various biological processes including cell differentiation, growth and development, stress responses, and disease occurrence and development. Thus, SOX4 may play an important role in the progression of NPC [20,21].

The objective of this study was to assess the role of HOTAIR in DDP-resistant NPC, and to understand the mechanisms underlying cell proliferation, invasion, and apoptosis. And this study aimed to explore the role of miR-106a-5p/SOX4 axis in NPC drug resistance and to demonstrate the central role played by HOTAIR. It is significant for looking for novel therapeutic targets in the treatment of NPC drug resistance.

## 2. Materials and methods

### 2.1. Patient methods

We collected the tumor tissues surgically removed from all patients and selected 10 cases of DDP-sensitive and 10 cases of DDP-resistant tissues according to the Huvos scoring system at The Second Hospital of Anhui Medical University between 2014 and 2018. Participation was required to sign written informed consent. Proof of the ethical approvals was verified by the local Ethics Committee (SL-YX2019-061).

### 2.2. Cell culture and transfection

C666-1 and CNE2 cell lines were purchased from the American Type Culture Collection (Manassas, VA, USA). C666-1 and CNE2 cells were selected and used to construct C666-1/DDP and CNE2/DDP cells (DDP-resistant NPC cell lines) via intermittent shock and an increasing concentration gradient over 6 months [22,23]. The cells were cultured at a density of 5 × 10^5^/mL in Mccoy’s 5A medium containing 10% fetal bovine serum and 1% penicillin-streptomycin-glutamine. HOTAIR small interfering RNA (siRNA), SOX4 siRNA, and the miR-106a-5p inhibitor were synthesized by GenePharma (Shanghai, China) and transfected into cells with Lipofectamine 2000 (Invitrogen, Carlsbad, CA, USA). The forward and reverse primer sequences were as follows: si-HOTAIR, 5’-GCA CAG AGA AAU GGC AAA UU-3’ and 5’-UUU GCC AUU AUC UCU GUG CUU-3’; si-SOX4, 5’- UUC UCC GAA CGU GUC ACG UTT-3’ and 5’- ACG UGA CAC GUU CGG AGA ATT-3’; and miR-106a-5p inhibitor, 5’- CUA CCU GCA CUG UAA GCA CUU UU-3’.

### 2.3. Apoptosis assay

Apoptosis analysis was performed by using flow cytometer. Cells were collected and washed with PBS. Then resuspended (3 × 10^3^ cells/well) in 500 μL binding buffer and stained with 5 μL annexin V-fluorescence isothiocyanate and 10 μL propidium iodide for 5–15 min at room temperature. Flow cytometry was used to detect cell apoptosis [24].

### 2.4. Cell counting Kit-8 assays

The cells were seeded (2 × 10^3^ cells/96 well) and cultured in a 100 μL medium containing 10% FBS for 72 h. Then, 10 μL CCK-8 solution was added to each well of the plate for 2-h incubation, and the absorbance was measured at 490.

### 2.5. Quantitative RT-PCR

Total RNA was extracted using Trizol reagent (Invitrogen). The extracted RNA was quantified and reversely transcribed into complementary deoxyribose nucleic acid (cDNA), followed by PCR using SYBR Green method. The relative level was calculated by 2 ^−ΔΔCT^ method.

### 2.6. Transwell assay

The migration and invasion of cells were measured using Transwell plates (pore filter size, 8 µm; Corning Inc., Corning, NY, USA). The Transwell filter chamber was pre-coated with 50 mg/L Matrigel at a ratio of 1:8 at 4°C. After incubation for 24 h, cells (5,000 cells /well) on the upper chamber surface were removed, and cells adhering to the bottom side of the Transwell insert were stained with 0.1% crystal violet and counted.

### 2.7. Luciferase assay

The wild-type HOTAIR-3’-UTR (WT-HOTAIR-3’-UTR) or SOX4-WT (WT-SOX4-3’-UTR) and mutant HOTAIR-3’-UTR (MUT-HOTAIR-3’-UTR) or SOX4-MUT (MUT-SOX4-3’-UTR) containing the miR-106a-5p binding sites were cloned into the firefly luciferase-expressing psicheck2 vector (Promega, WI, USA) for luciferase reporter experiments and then co-transfected into cells. Luciferase assay was performed the firefly luciferase 48 h post-transfection.

### 2.8. Western blotting

Total protein was extracted from samples using radioimmunoprecipitation assay (RIPA) and determined the concentration of protein using a bicinchoninic acid assay kit (Sigma-Aldrich; Merck KGaA, Darmstadt, Germany). Then 50 μg proteins were separated by 10% SDS-PAGE (100 V, 2 h) and electro-transferred onto a polyvinylidene difluoride (PVDF) membrane (Millipore, Boston, MA, USA) [25]. Membranes were blocked with 5% bovine serum albumin (Thermo Fisher Scientific, Inc.) for 2 h at room temperature, then incubated with primary antibodies overnight at 4°C.The primary antibodies of MDR1 (ab170904) (1:1000), MRP5 (ab230674) (1:1000), LRP1 (ab92544) (1:1000), and β-actin (1:3000) were purchased from Abcam Cambridge, MA, USA, ABCB1 (12–2439-42) (1:1000) was purchased from Invitrogen, USA. Then, the membranes were incubated with horseradish peroxidase-conjugated secondary antibodies (Santa Cruz) for 1 h at room temperature. The signals were detected by ECL method.

### 2.9. RNA immunoprecipitation

To further confirm the target, RNA immunoprecipitation was performed with RNA Immunoprecipitation Kit (Guangzhou Geneseed biotech, Co.Ltd, China) as instructed. 1 × 10^5^ cells at 75% confluence were transfected. At posttransfection of 48 h, the cell was lysed in RIP lysis buffer. The supernatant was prepared through centrifugation and incubated with 10 µL anti-Ago2 antibodies and normal IgGs at 4°C for 2 h following another 2 h incubation with 40 µL protein A/G beads. Successful immunoprecipitation was evaluated by RT-qPCR.

### 2.10. *Tumor xenograft in* vivo

BALB/c nude mice were used to divided to five groups [1]: NC group (injected with the C666-1/DDP and CNE2/DDP cell lines) [2], si-HOTAIR (injected with the C666-1/DDP and CNE2/DDP cell lines with HOTAIR knockdown) [3], miR-106a-5p inhibitor group (injected with the C666-1/DDP and CNE2/DDP cell lines with miR-106a-5p knockdown) [4], si-SOX4 group (injected with the C666-1/DDP and CNE2/DDP cell lines with SOX4 knockdown) [5], si-HOTAIR+ miR-106a-5p inhibitor group (injected with the C666-1/DDP and CNE2 /DDP cell lines with both HOTAIR and miR-106a-5p knockdown). cell suspension containing 2 × 10^6^ cells was subcutaneously injected into the left back of each mouse. The digital caliper was used to assess length (l) and width (w) of tumor tissues and calculating the volume (V) as follows: V = lw^2^/2. After 28 days, the mice were anesthetized with 3% pentobarbital sodium (150 mg/kg body weight) and tumor tissues were collected and weighted.

### 2.11. Statistical analysis

Values are expressed as mean ± standard deviation (mean ± SD). Student t-test was used to indicate the difference between two groups while one-way ANOVA was performed for the difference between three or multiple groups. *P* < 0.05 indicated statistical significance.

## 3. Results

Our results demonstrated that HOTAIR was overexpressed in DDP-resistant NPC tissues and cells compared with normal tissue and cells. Inhibition of HOTAIR expression decreased the resistance of NPC cells to DDP by mediating miR-106a-5p/SOX4 axis activity, mainly shown as suppressing cell viability, invasion and migration, and promoting apoptosis in DDP-resistant NPC cells.

### 3.1. Down-regulation HOTAIR expression decreases the resistance of NPC cells to DDP

To investigate the effects of HOTAIR on DDP resistance in NPC, HOTAIR expression levels were measured using qPCR. The results showed that HOTAIR expression levels were significantly increased in NPC tissues, especially DDP-resistant tissues compared with normal tissues (Supplementary Fig.S1A). Also, HOTAIR expression levels were also upregulated in DDP-resistant NPC cells compared with their normal cell lines (Supplementary Fig.1SB).

**Figure 1.**
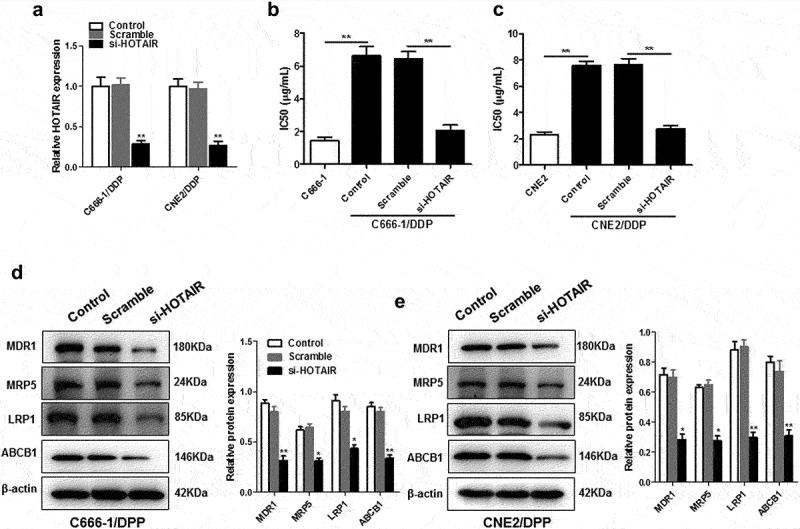
Down-regulation of HOTAIR decreased the resistance of C666-1/DDP and CNE2/DDP cells to DDP. HOTAIR siRNA was transfected into C666-1/DDP and CNE2/DDP cells, following transfection for 48 h, the interference efficiencies were detected with qPCR (a). The IC_50_ values of DDP (b, c), the protein levels of MDR1, MRP5, LRP1and ABCB1 (d, e) were detected by Western blotting. *p < 0.05, **p < 0.01.

As shown in Figure 1(a), si-HOTAIR was effectively transfected into C666-1/DDP or CNE2/DDP cells (Figure 1(a)), which led to a significant decrease in IC_50_ values (Figure 1(b,c)). Knockdown of HOTAIR in C666-1/DDP and CNE2/DDP cells reduced the expression of chemoresistance-related protein including multidrug resistance mutation 1 (MDR1), multidrug resistance-associated protein 5 (MDR5), LDL receptor-related protein-1 (LRP1), and ATP-binding cassette subfamily B member 1 (ABCB1) (Figure 1(d,e)).

### 3.2. Downregulation of HOTAIR suppresses cell viability, invasion and migration, and promotes apoptosis in DDP-resistant NPC cells

**Figure 2.**
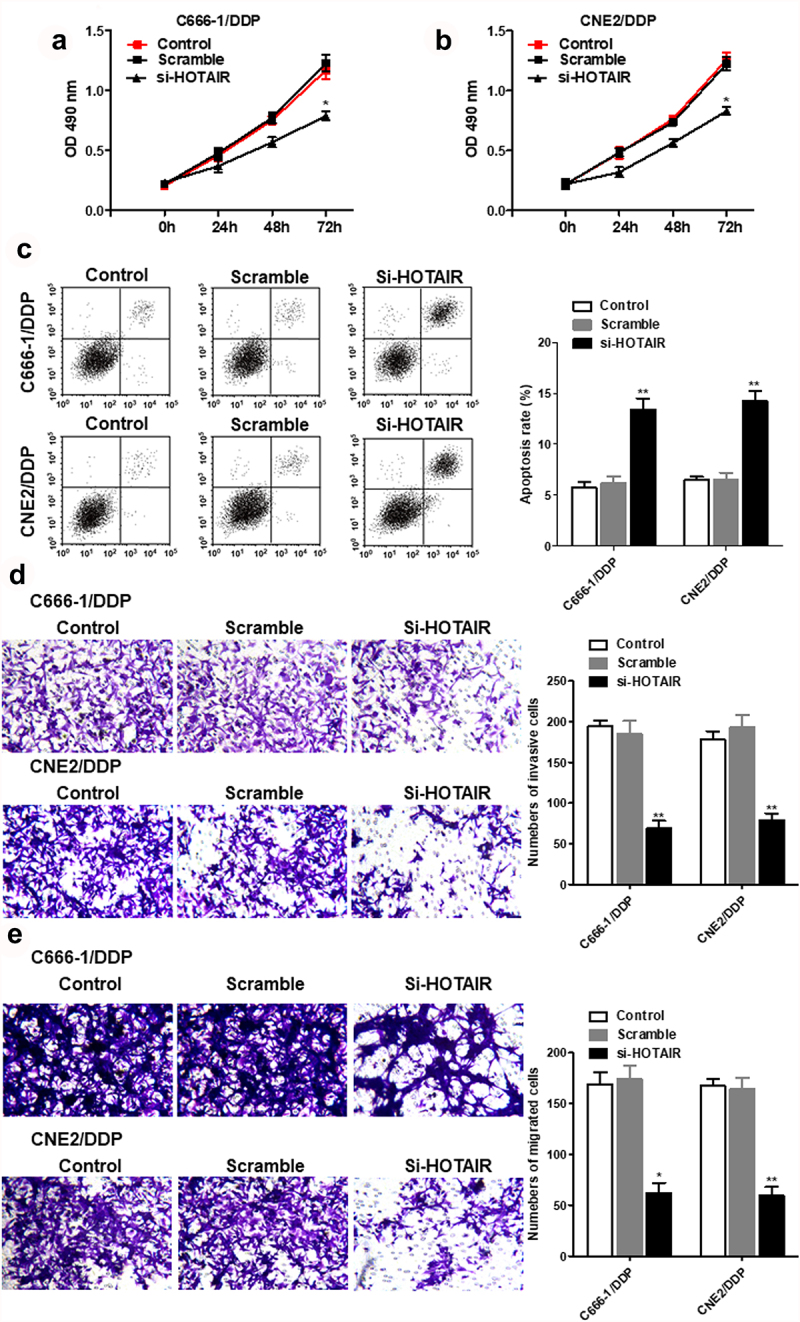
Interference with HOTAIR inhibited proliferation and invasion, and promoted apoptosis of C666-1/DDP and CNE2/DDP cells. HOTAIR siRNA was transfected into C666-1/DDP and CNE2/DDP cells, following transfection for 48 h, the cell proliferation (a, b), apoptosis (c), invasion and migration (d, e) were detected by CCK-8, AnnexinV/PI and Transwell *p < 0.05, **p < 0.01.

Si-HOTAIR inhibited cell viability in C666-1/DDP and CNE2/DDP cells, migration and invasion in DDP-resistant NPC cells. But the apoptosis was increased in si-HOTAR transfected DDP-resistant NPC cells (Figure 2(a-e)).

### 3.3. Low expression of miR-106a-5p in DDP-resistant NPC tissues and cells and downregulated HOTAIR promotes miR-106a-5p expression

**Figure 3.**
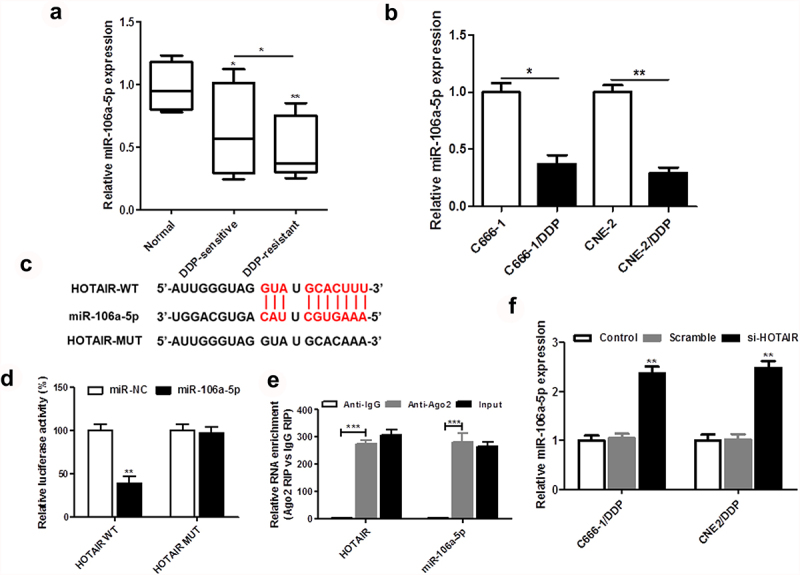
miR-106a-5p was downregulated in DDP-resistant NPC tissues and cells and regulated by HOTAIR. The expression of HOTAIR in DDP-sensitive and DDP-resistant NPC tissues (a), C666-1/DDP and CNE2/DDP cells and their matched controls were measured by qPCR (b). (c) The binding site of HOTAIR and miR-106a-5p predicted by StarBase v2.0. (d) The Luciferase activities of HOTAIR-WT (HOTAIR-MUT) reporters in C666-1/DDP and CNE2/DDP cells co-transfected with miR-106a-5p mimic or NPC mimic were assessed by Dual-Luciferase reporter assay. (e) RIP assay was performed to observe the position relation between HOTAIR and miR-106a-5p (f) HOTAIR siRNA was transfected into C666-1/DDP and CNE2/DDP cells, following transfection for 48 h, the expression of miR-106a-5p were detected by qPCR. *p < 0.05, **p < 0.01.

**Figure 4.**
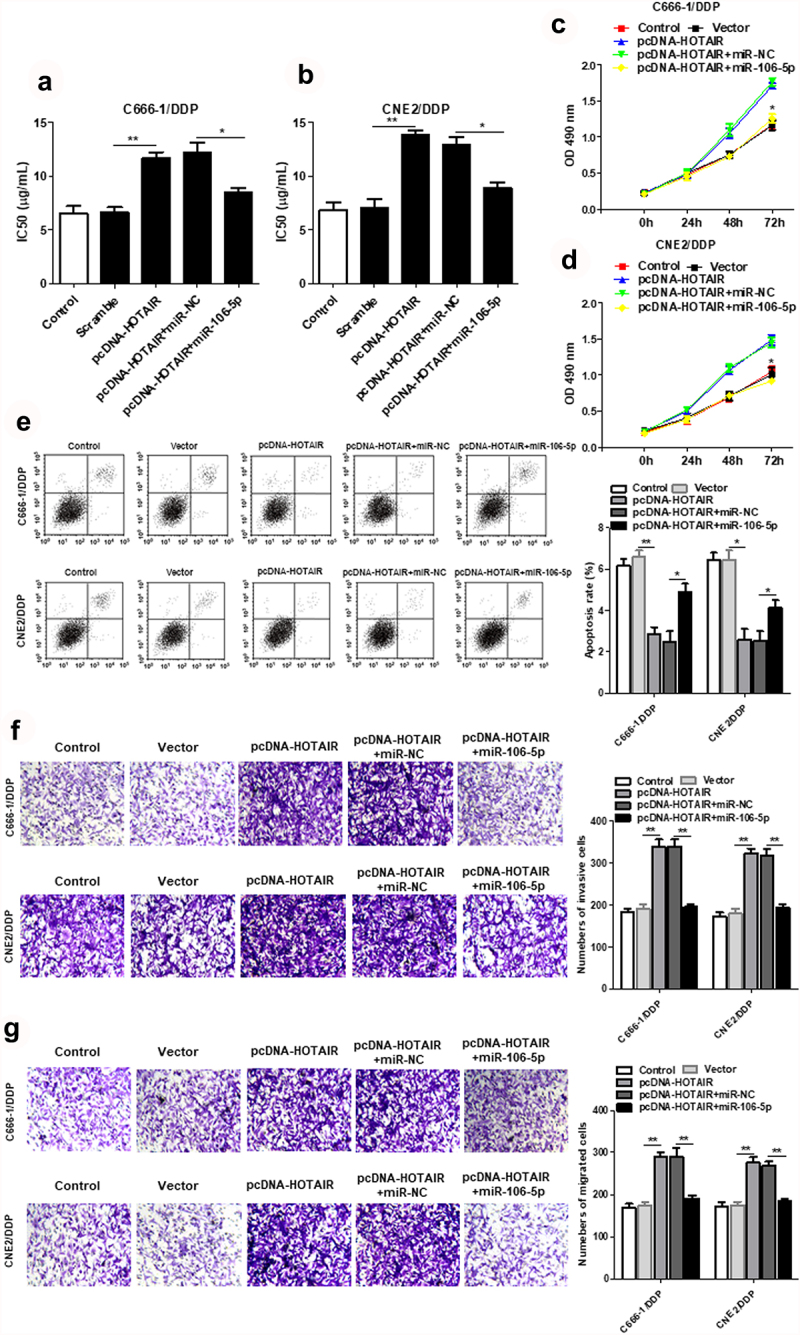
HOTAIR regulated the DDP resistance, cell proliferation, invasion and apoptosis of C666-1/DDP and CNE2/DDP cells by binding miR-106a-5p. HOTAIR overexpression vector was transfected into C666-1/DDP and CNE2/DDP cells alone or together with miR-106a-5p mimic, following transfection for 48 h, the IC50 values of DDP (a, b), the cell proliferation were detected by CCK-8 (c, d), and cell apoptosis (e), invasion (f) and migration (g) were detected by AnnexinV/PI and Transwell. *p < 0.05, **p < 0.01.

Our results showed that miR-106a-5p was markedly decreased in DDP-resistant NPC tissues and cells (Figure 3(a,b)). As shown in Figure 4(c), miR-106a-5p was determined to be the target site of HOTAIR. The results of dual luciferase reporter assay were confirmed that HOTAIR was the target of miR-106a-5p using wild-type and mutant-binding sites (Figure 3(d)). RIP assay showed the significant enrichment of miR-106a-5p and HOTAIR using Ago2 antibody compared with IgG antibody (Figure 3(e)). In addition, decreased HOTAIR expression dynamically increased the miR-106a-5p expression in DDP-resistant NPC cells (figure 3(f)).

### 3.4. HOTAIR binds miR-106a-5p and regulates the DDP resistance, cell viability, invasion and apoptosis of DDP-resistant NPC cells

HOTAIR overexpression increased the IC_50_ values of C666-1/DDP and CNE2/DDP cells, while increased miR-106a-5p decreased the IC_50_ values (Figure 4(a,b)). Meanwhile, up-regulated miR-106-5p expression reduced cell viability, which was promoted by HOTAIR overexpression (Figure 4(c,d)). Additionally, miR-106a-5p mimic promoted the cell apoptosis induced by overexpression of HOTAIR (Figure 4(e)). We also confirmed that upregulated miR-106-5p suppressed the cell invasion and migration induced by overexpression of HOTAIR (figure 4(f,g)).

### 3.5. SOX4 targets miR-106a-5p

**Figure 5.**
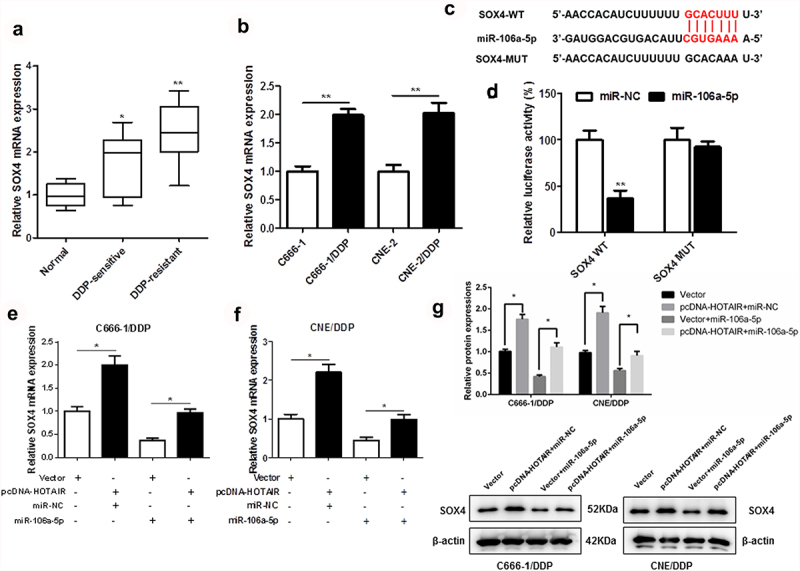
SOX4 was a target of miR-106a-5p. The expression of SOX4 in DDP-sensitive and DDP-resistant NPC tissues, C666-1/DDP and CNE2/DDP cells and their matched controls were measured by qPCR (a, b). Binding sites of miR-106a-5p and SOX4 predicted by TargetScan Human 7.2 (c). The Luciferase activities of SOX4-WT (SOX4-MUT) reporters in C666-1/DDP and CNE2/DDP cells transfected with miR-106a-5p mimic or NPC mimic were assessed by Dual-Luciferase reporter assay (d). HOTAIR overexpression vector and miR-106a-5p mimic were transfected respectively or co-transfected into C666-1/DDP and CNE2/DDP cells, and the expression of SOX4 was detected by qPCR (e, f). HOTAIR overexpression vector and miR-106a-5p mimic were transfected respectively or co-transfected into C666-1/DDP and CNE2/DDP cells, and the expression of SOX4 was detected by Western blot (g). *p < 0.05, **p < 0.01.

As shown in Figure 5(a) and (b), the mRNA expression of SOX4 in DDP-resistant NPC tissues and cells was upregulated. We found that SOX4 3ʹUTR had two binding sites with miR-106a-5p sequence (Figure 5(c)). In addition, dual-luciferase reporter assay also confirmed the targeted relation between SOX4 and miR-106a-5p (Figure 5(d)). Moreover, HOTAIR overexpression significantly increased the mRNA expression (Figure 5(e) and (f)) protein levels (Figure 5(g)) of SOX4, which was inhibited by miR-106a-5p in DDP-resistant NPC cells.

### 3.6. HOTAIR mediated miR-106a-5p/SOX4 axis activity to regulate DDP resistance, cell viability, invasion and apoptosis in NPC DDP-resistant cells

**Figure 6.**
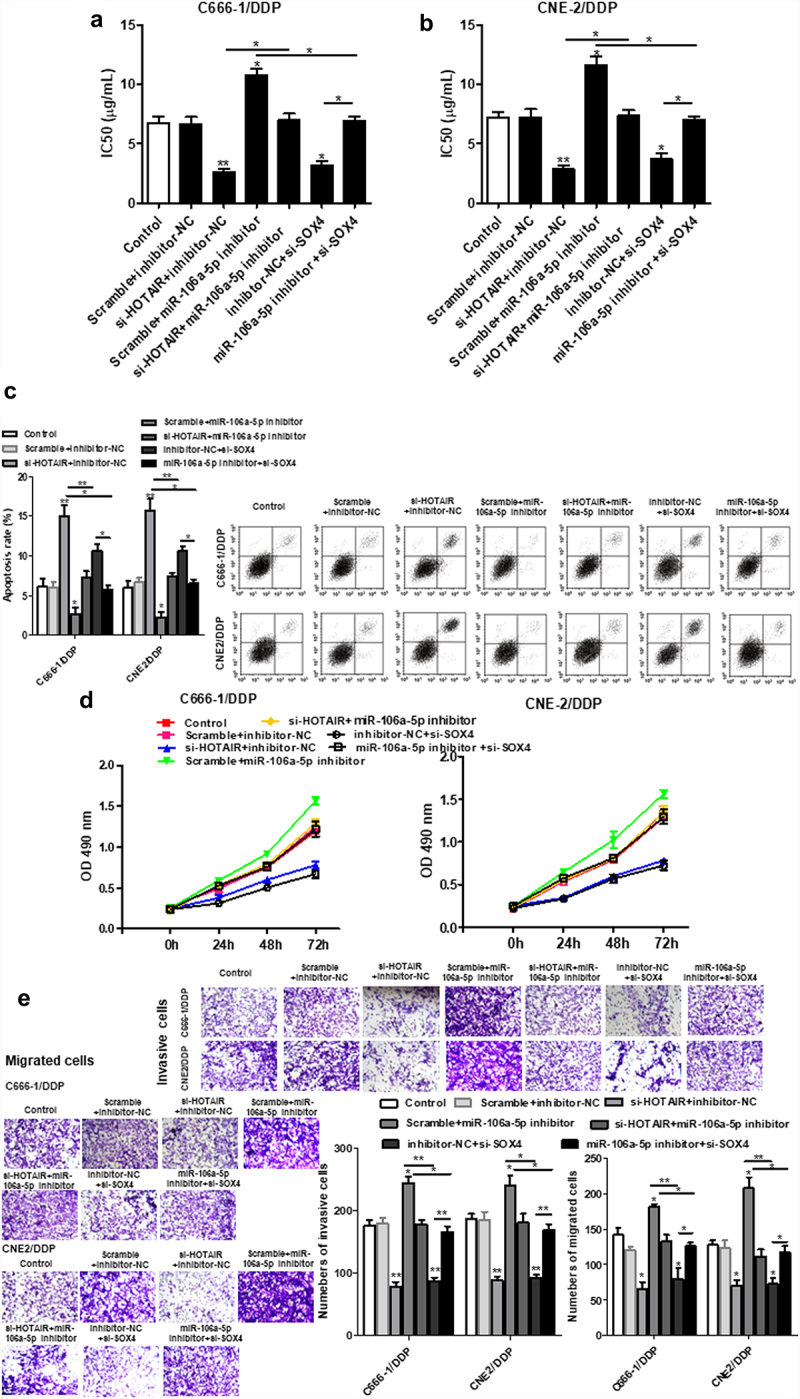
HOTAIR regulated the DDP resistance, apoptosis, cell proliferation, invasion and migration of C666-1/DDP and CNE2/DDP cells via miR-106a-5p/SOX4 axis. The HOTAIR siRNA, miR-106a-5p inhibitor, HOTAIR siRNA + miR-106a-5p inhibitor, SOX4 siRNA, miR-106a-5p inhibitor+ SOX4 siRNA were transfected into C666-1/DDP and CNE2/DDP cells, following transfection for 48 h, the IC50 values of DDP (a, b). The apoptosis was determined by AnnexinV/PI (c). Cell proliferation (d), invasion and migration (e) were detected by CCK-8 and Transwell. *p < 0.05, **p < 0.01.

Further to explore the relationship of the miR-106a-5p/SOX4 axis with HOTAIR in NPC DDP-resistant cells, we found that si-SOX4 inhibited the increased IC_50_ values induced by miR-106a-5p inhibitors (Figure 6(a,b)), as well as the increased apoptosis (Figure 6(c)). miR-106a-5p inhibitor could increase the invasion and migration of cells, and si-SOX4 increased those effects in NPC DDP-resistant cells (Figure 6(d,e)).

### 3.7. *HOTAIR/miR-106a-5p/SOX4 axis modulates NPC DDP-resistant tumor growth* in vivo

**Figure 7.**
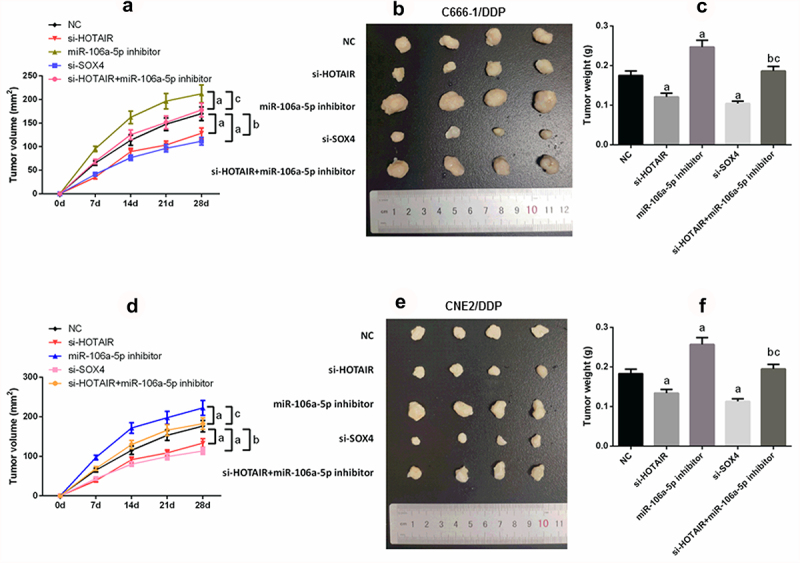
HOTAIR/miR-106a-5p/SOX4 axis mediated NPC DDP resistance tumorgenesis in vivo. (a–c) Suppression of tumor growth was observed after HOTAIR or SOX4 knockdown in C666-1/DDP cell line; (d–f) Suppression of tumor growth was observed after HOTAIR or SOX4 knockdown in CNE2/DDP cell line. Tumor size was enlarged by transfecting with miR-106a-5p inhibitor, tumor volume was retrieved in si-HOTAIR group on addition of miR-106a-5p inhibitor. Tumor growth was measured every other day after 7 days of injection, and tumors were then harvested on day 28 and weighed. Actual tumor size after the harvest was shown in the medium panel. a compared with NC group, *p* < 0.05. b compared with si-HOTAIR group, *p* < 0.05. c compared with miR-106a-5p-inhibitor group, *p* < 0.05.

In order further to determine the role of HOTAIR/miR-106a-5p/SOX4 axis in NPC DDP-resistant, the results of nude mice experiment showed that silencing HOTAIR or si-SOX4 decreased tumor growth, while the miR-106a-5p inhibitor promoted tumor growth (P < 0.05; Figure 7(a–f)). Meanwhile, si-HOTAIR combined miR-106a-5p inhibitor showed more anti-tumor growth.

## 4. Discussion

The most significant shortcoming of DDP therapy is that tumor cells are prone to becoming drug-resistant [26,27]. There are two main forms of DDP resistance: inherent and acquired resistance [28]. The mere elaboration of the mechanism of NPC resistant to DDP alone has important therapeutic value. In this study, we aimed to detect the role of HOTAIR in DDP resistance in NPC, and the underlying molecular mechanisms.

LncRNAs (more than 200bp in length) are most highly conserved in sequence, which constructs an intricate network of interplays with various diverse biomolecules (DNA, RNA or protein), and thusly their perturbation exhibits profound regulatory influences and plays a new role on cancer [29]. Dysfunction of lncRNA is closely associated with the occurrence and development of numerous diseases, including cancer [30]. HOTAIR can regulate the resistance of lung cancer cells to DDP by reducing the expression of p21WAF1/CIP1 [31]. In addition, HOTAIR activates the PI3K/AKT/MRP1 signaling pathway by targeting miR-126, to promote DDP resistance in gastric cancer [32]. In this study, we observed increased HOTAIR expression in NPC tissue, especially DPP-resistant tissues. Interestingly, HOTAIR expression was increased in DPP-resistant NPC cells. Our results showed that knockdown of HOTAIR could significantly downregulate DDP resistance, accompanied by decreased resistance to the expression of related genes in DDP-resistant NPC cells. Silencing HOTAIR expression decreased cell viability, migration and invasion in DDP-resistant NPC cells, and upregulated cell apoptosis.

MiRNAs have been identified as important posttranscriptional regulators involved in various biological and pathological processes of cells in tumor with DDP-resistance [33,34]. The expression of miR-26b significantly inhibits Jagged canonical notch ligand 1 expression in DDP-resistant NPC [35]. In addition, miR-106a-5p has been shown to regulate Fas-activated serine/threonine kinase, which has suppressive effects on astrocytoma [36]. Moreover, miR-106a-5p targets high mobility group AT-hook 2 and inhibits osteosarcoma progression [37]. Here, we also observed the decreased miR-106a-5p expression in DDP-resistant NPC tissues and cells. Moreover, miR-106a-5p mimics suppressed the DDP resistance induced by HOTAIR up-regulation. SOX4 was determined to be the target of miR-106a-5p, and silencing of SOX4 increased DDP sensitivity in DDP-resistant NPC cells.

In conclusion, this study demonstrates the role of HOTAIR in DDP-resistant NPC, and this study explores the role of the miR 106a-5p/SOX4 axis in drug-resistant NPC.

## 5. Conclusion

Taken together, our results revealed increased expression of HOTAIR and SOX4, and decreased miR-106a-5p levels, in DDP-resistant NPC tissues and cells. Inhibition of HOTAIR expression decreased the resistance of NPC cells to DDP by mediating miR-106a-5p/SOX4 axis activity, mainly shown as suppressing cell viability, invasion and migration, and promoting apoptosis in DDP-resistant NPC cells.

## Supplementary Material

Supplemental Material

## Data Availability

The data used to support the findings of this study are included in the article.
