# Supplementary figures and images for: HOTAIR mediates cisplatin resistance in nasopharyngeal carcinoma by regulating miR-106a-5p/SOX4 axis
Source: Bioengineered. 2022 Feb 28;13(3):6567–78. doi: 10.1080/21655979.2022.2038429 (PMC8975274; doi:10.1080/21655979.2022.2038429)

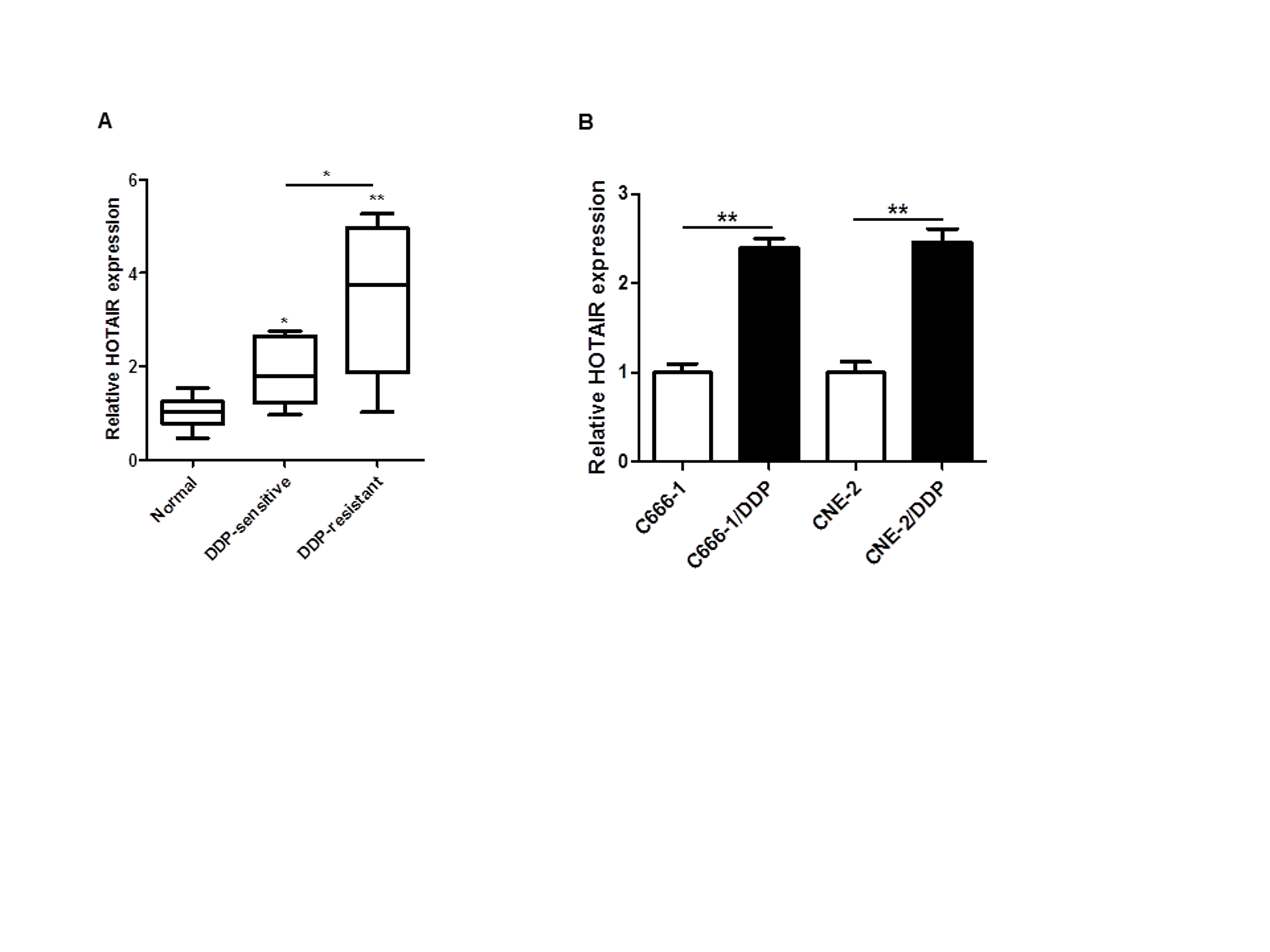

Supplement: Supplemental Material [file KBIE_A_2038429_SM4202.tif]
